# Modeling Liver Fibrosis Progression in Patients With Viral Hepatitis Using the Machine Learning Tool Subtype and Stage Inference (SuStaIn)

**DOI:** 10.7759/cureus.78744

**Published:** 2025-02-08

**Authors:** Akiyoshi Suzuki, Katsuhiro Sano, Yuya Saito, Peter Wijeratne, Kotaro Yamamoto, Shohei Fujita, Jun Woo, Nobuo Tomizawa, Koji Kamagata, Hiroshi Imamura, Shuichiro Shiina, Akio Saiura, Kenichi Ikejima, Ryohei Kuwatsuru, Yoshitaka Masutani, Daniel Alexander, Shigeki Aoki

**Affiliations:** 1 Department of Radiology, Juntendo University Graduate School of Medicine, Tokyo, JPN; 2 Department of Informatics, University of Sussex, Sussex, GBR; 3 Department of Radiology, Kokura Memorial Hospital, Kitakyushu, JPN; 4 Department of Radiology, The University of Tokyo, Tokyo, JPN; 5 Department of Radiology, The Jikei University School of Medicine, Tokyo, JPN; 6 Department of Hepatobiliary‐Pancreatic Surgery, Juntendo University Graduate School of Medicine, Tokyo, JPN; 7 Department of Gastroenterology, Juntendo University Graduate School of Medicine, Tokyo, JPN; 8 Division of Health Sciences, Tohoku University Graduate School of Medicine, Sendai, JPN; 9 Centre for Medical Image Computing, Department of Computer Science, University College London, London, GBR

**Keywords:** cirrhosis, hepatitis b virus, hepatitis c virus, liver fibrosis, subtype and stage inference, sustain

## Abstract

Background and aim

The natural progression of liver fibrosis and its association with biomarker changes have not been fully established in the literature. This study aimed to investigate liver fibrosis progression in patients with hepatitis B virus (HBV) and hepatitis C virus (HCV) infection using a novel machine learning tool called ‘Subtype and Stage Inference (SuStaIn).’ SuStaIn can identify disease progression patterns and subgroups from cross-sectional biomarker data.

Methods

This single-center retrospective study included 168 consecutive patients (mean age, 67.0 years ± 13.2; 91 male), comprising 29 with HBV, 50 with HCV, and 89 controls. All patients underwent gadoxetic acid-enhanced magnetic resonance imaging between January 2019 and December 2019. Imaging biomarkers measured were quantitative liver-spleen contrast ratio (Q-LSC), contrast index of liver-muscle signal intensity (CEI), and right liver lobe to the total liver volume ratio (RV/TV), while the serum biomarkers were fibrosis-4 index (FIB-4) and aspartate aminotransferase to platelet ratio index (APRI). SuStaIn was applied using z-scored biomarkers derived from each biomarker relative to the control group.

Results

The most likely ordering of the biomarker conversion in liver fibrosis progression was determined to be APRI, FIB-4, CEI, Q-LSC, and RV/TV. This sequence was observed to be consistent in all patients infected with either HBV or HCV, regardless of the virus type.

Conclusions

Using SuStaIn, new insights into liver fibrosis progression in patients infected with either HBV or HCV were achieved: abnormalities in serum biomarkers occurred first, followed by a decrease in hepatic uptake of gadoxetic acid, ultimately resulting in right lobe atrophy.

## Introduction

Liver fibrosis is a chronic progressive pathological condition resulting from various causes, such as alcohol-associated liver disease, metabolic dysfunction-associated steatohepatitis (MASH), autoimmune diseases, and viral hepatitis. In particular, hepatitis B virus (HBV) and hepatitis C virus (HCV), which are prevalent worldwide and are major contributors to liver fibrosis, cause chronic hepatocyte injury and repair, fibrous tissue deposition in portal and interlobular areas, nodular regeneration, and ultimately, cirrhosis. Cirrhosis, the terminal stage of liver fibrosis, significantly increases the risk of hepatocellular carcinoma and manifests with various symptoms associated with portal hypertension, such as ascites, variceal bleeding, and hepatic encephalopathy.

Liver biopsy is the reference standard for diagnosing liver fibrosis and is the most specific test for assessing the nature and severity of liver diseases [1]. Various staging systems for liver fibrosis based on pathological findings are commonly used in clinical practice [2,3]. Accurate and timely evaluation of the extent of liver fibrosis is crucial for understanding its pathophysiology, predicting prognosis, and determining therapeutic strategies. However, liver biopsy has several disadvantages, including invasiveness, sampling errors, inter-rater diagnostic discrepancies, and high cost [4-7]. Due to these limitations, various noninvasive biomarkers have been developed for estimating liver fibrosis and are increasingly being used in place of liver biopsy [8-12]. Although these noninvasive biomarkers correlate with liver fibrosis staging systems, the natural history of liver fibrosis has not yet been well established, and the ordering of these biomarker conversions remains unclear.

Recent advancements in machine learning, particularly in disease progression modeling, have allowed the reconstruction of long-term pathology from cross-sectional data. Fonteijn et al. introduced the ‘event-based model’ [13], which initiated a series of unsupervised machine learning methods, collectively referred to as ‘Disease Progression Modeling’ [14]. This method reconstructs long-term temporal disease progression from cross-sectional data and, to date, has been applied mainly in the field of neurodegenerative disease. A key innovation was introduced by Young et al., who developed a novel technique known as 'Subtype and Stage and Inference' (SuStaIn). This machine-learning tool identifies disease subgroups and progression patterns from cross-sectional biomarker data by combining disease progression modeling and clustering [15-18].

Therefore, we hypothesized that applying this technique could identify disease-specific liver fibrosis progression patterns and aimed to identify the subtypes of liver fibrosis progression in patients infected with either HBV or HCV using SuStaIn.

## Materials and methods

Patients

This retrospective study was approved by the Institutional Review Board of Juntendo University Hospital (IRB No. H20-0197). The requirement for written informed consent was waived due to the retrospective nature of the study. We screened 300 consecutive patients with known or suspected chronic liver disease with focal liver lesions who underwent gadoxetic acid-enhanced MRI between January 2019 and December 2019. 

**Figure 1:**
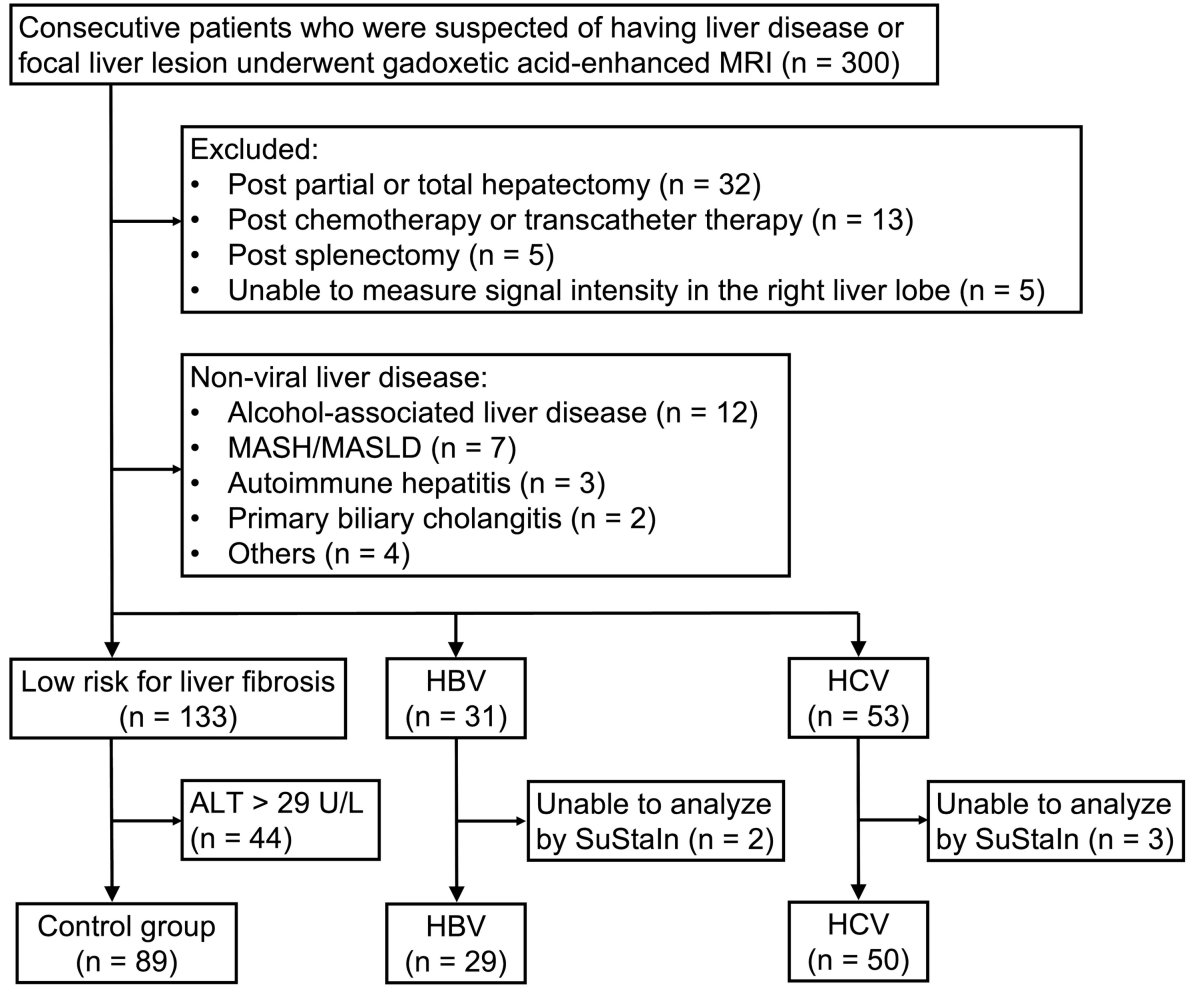
Patient flowchart MASH: metabolic dysfunction-associated steatohepatitis; MASLD: metabolic dysfunction-associated steatotic liver disease; HBV: hepatitis B virus; HCV: hepatitis C virus; ALT: alanine aminotransferase. The image is created by the author.

A selection flowchart is shown in Figure 1. The exclusion criteria were as follows: post-partial and total hepatectomy (n=32), previous chemotherapy and transcatheter therapy (n=13), post-splenectomy (n=5), inability to measure signal intensity in the right liver lobe (n=5), and non-viral liver disease (n=28). The study population comprised patients with HBV (n=31), HCV (n=53), and low-risk for liver fibrosis (n=133). Although SuStaIn requires a normal control group, it is impractical to use contrast agents in healthy volunteers. Therefore, we defined the low-risk liver fibrosis group as patients without chronic liver disease, such as viral hepatitis. This group included not only patients in a stable state, who could serve as normal controls but also those with transient liver enzyme elevations due to conditions like biliary inflammation. To better define the normal control group and avoid underestimating the disease group, we excluded patients with alanine aminotransferase (ALT) levels >29 U/L (n=44) from the low-risk for liver fibrosis group, following a previous study [19]. Finally, 168 individuals (mean age, 67.0 ± 13.2 years; 91 male), comprising those with HBV (n=29) and HCV (n=50) and controls (n=89), were enrolled in this study. Later, three patients with HBV and two patients with HCV were excluded due to the inability to analyze their data using SuStaIn.

Magnetic resonance imaging

Gadoxetic acid-enhanced MR imaging was performed using a 1.5-T and 3.0-T superconducting system (Ingenia 1.5-T [n=92], Achieva 1.5-T [n=28]: Philips Medical Systems, Amsterdam, the Netherlands; Avanto 3.0-T [n=82], Skyra 3.0-T [n=21], Prisma 3.0-T [n=12]: Siemens Healthcare, Forchheim, Germany; Galan 3.0-T [n=65]: Canon Medical Systems, Tochigi, Japan) equipped with an 8-32-channel body coil. Pre-contrast fat-saturated T1-weighted gradient-echo images and hepatobiliary phase images were obtained with a three-dimensional acquisition sequence using the following parameters: repetition time (msec)/echo time (msec), 2.6-4.0/1.1-2.1; flip angle, 9-15°; field of view, 350-380 × 270-360 mm^2^; image matrix, 224-320 × 156-224; section thickness, 3 mm with a 0-mm overlap or 5 mm with a 2.5-mm overlap; acquisition time, 17-21 s. Gadoxetate contrast material (Primovist; Bayer Healthcare [Leverkusen, Germany]) was administered intravenously as a bolus at a dose of 0.1 ml/kg of body weight with a fixed duration of 4 seconds, followed by 30 mL of saline using a power injector (Sonic Shot 7; Nemoto Kyorindo, Tokyo, Japan). Hepatobiliary phase images were obtained 20 min post-injection. The acquired images were transferred to a workstation for post-processing (Synapse Vincent, version 6.0; Fujifilm Medical, Lexington, Massachusetts), where right and total liver lobe volumes were measured. 

Imaging biomarkers for liver fibrosis

**Figure 2:**
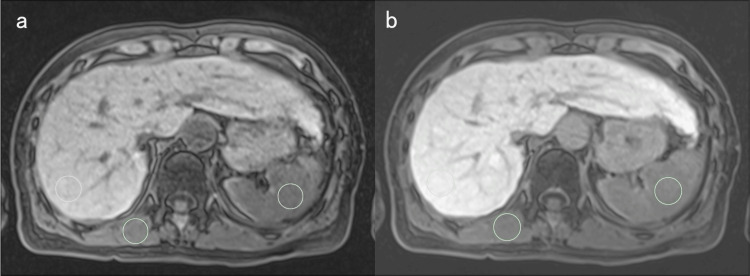
Signal intensity measurement Signal intensity measurement on pre-contrast fat-saturated T1-weighted gradient-echo images (a) and hepatobiliary phase images (b) in a female patient in her 60s from the HBV group. The regions of interest (green circles) were drawn on the parenchyma of the right liver lobe, spleen, and paraspinal muscle in the same axial image. The contrast index of the liver-muscle signal intensity and quantitative liver-spleen contrast ratio were calculated using these signal intensities.

The quantitative liver-spleen contrast ratio (Q-LSC) [8] and contrast index of liver-muscle signal intensity (CEI) [9,10], which reflected the decrease in hepatic uptake of gadoxetic acid, were used as the imaging biomarkers for liver fibrosis. Q-LSC was calculated as the ratio of the signal intensity of the liver parenchyma to that of the spleen 20 min post-contrast media administration. CEI was calculated by dividing the ratio of the signal intensity of the liver parenchyma to the paraspinal muscle in the hepatobiliary phase on MRI by the ratio of the signal intensity of the liver parenchyma to the paraspinal muscle in the pre-contrast phase. For signal intensity measurement, the regions of interest (ROI) were drawn on the parenchyma of the posterior segment of the right liver lobe, the center of the spleen, and the right paraspinal muscle within the same axial image using a commercially available picture archiving and communication system (SYNAPSE; Fujifilm Medical, Lexington, Massachusetts). Each ROI was within 20 mm in diameter depending on the size of the parenchyma (Figure 2).

**Figure 3:**
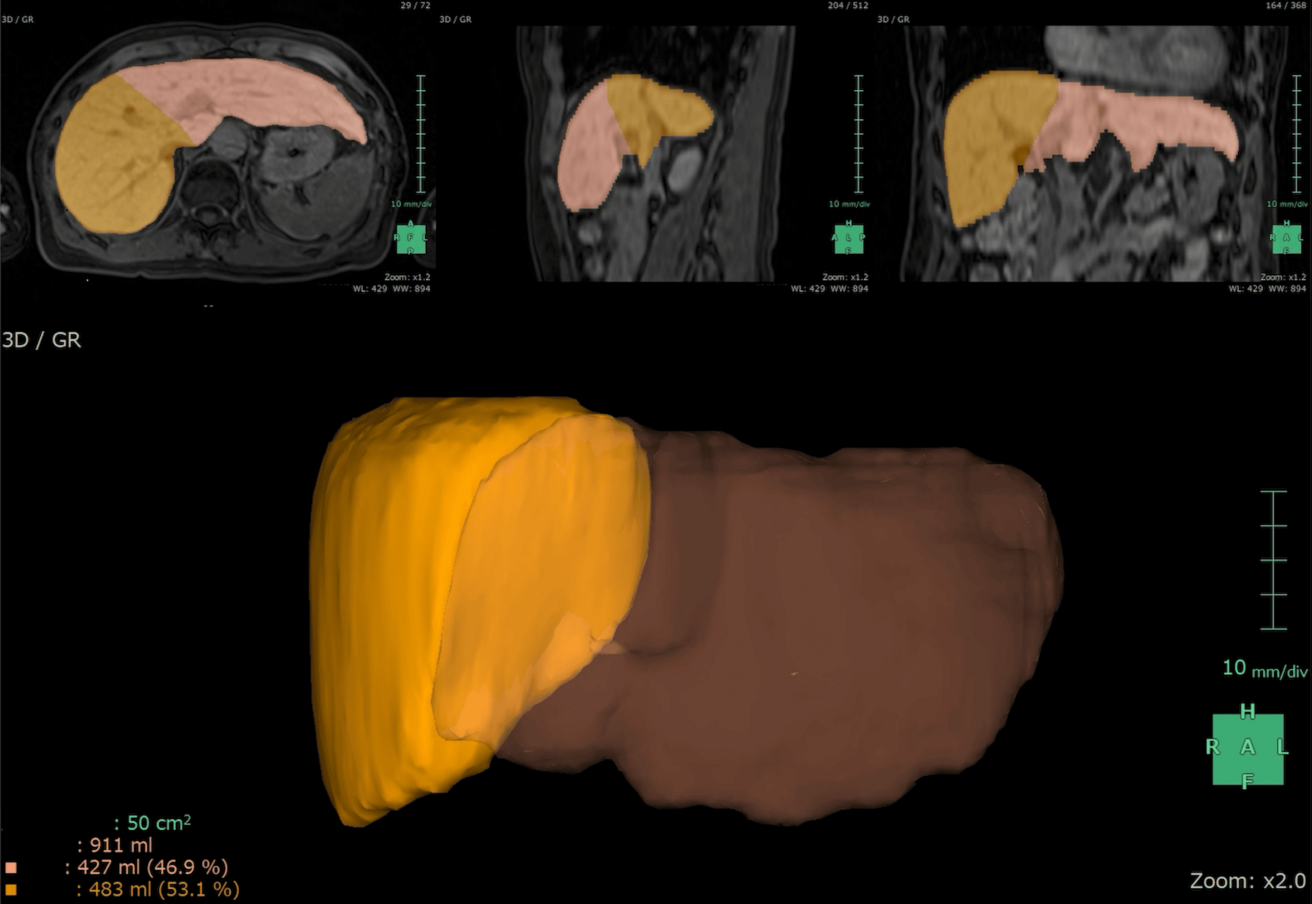
Volume measurement Right liver lobe volume to total liver volume ratio (RV/TV) measurement in a female patient in her 60s from the HBV group (same as Figure 2). Right liver lobe was depicted by manually drawing a boundary between the right and left liver lobe along the middle hepatic vein. In this case, RV/TV was automatically calculated as 0.53 (483 ml/911 ml) using a commercially available application.

In addition, the right liver lobe volume to total liver volume ratio (RV/TV) was used as one of the imaging biomarkers. The right liver lobe was determined by manually delineating the boundary between the right and left liver lobes along the middle hepatic vein and measured automatically using a commercially available application (Liver Analysis; Fujifilm Medical, Lexington, Massachusetts) (Figure 3). Total liver lobe volume was also measured using the application.

To assess reproducibility, we randomly selected 30% (50/168) of the total subjects and performed measurements independently by two examiners (radiologists with 4 and 20 years of post-training experience). The intraclass correlation coefficient (ICC) was calculated to evaluate inter-rater reliability. Since a high reproducibility was confirmed and the inter-rater variability was small (as detailed in the Results section), the remaining measurements were conducted by a single examiner (a radiologist with four years of post-training experience).

Serum biomarkers for liver fibrosis

The fibrosis-4 index (FIB-4) and aspartate aminotransferase-to-platelet ratio index (APRI) were calculated as serum biomarkers. FIB-4 was calculated as [age (years) × aspartate aminotransferase (AST) value (U/L)] / [platelet count (10^9^/L) × (ALT value (U/L))1/2] [11]. APRI was calculated as [AST value (U/L) / upper limit of normal AST value (U/L)] × 100 / platelet count (10^9^/L) [12]. These are noninvasive indexes that are widely used in clinical practice for predicting liver fibrosis due to their simplicity.

SuStaIn 

We implemented SuStaIn, an unsupervised machine learning tool designed to identify a set of disease subtypes and their progression patterns from cross-sectional biomarker data, in accordance with previous studies [17,18]. The technique simultaneously performed clustering and disease progression modeling. Each disease progression pattern was inferred as a sequential conversion of individual biomarker measurements from one z-score to another relative to a control population, where the control population had a mean of 0 and a standard deviation of 1. In this study, biomarker conversion was defined as the linear accumulation from 0 to 1 z-score. Since the imaging biomarkers used in this study decrease with disease progression, these z-scores become negative; we multiplied these z-scores by -1 so that z-scores increased as the imaging biomarkers decreased. Subtypes and disease stages were determined based on the highest likelihood of participants being assigned to each disease subtype and disease stage. To determine the optimal ordering, large amounts of orders were simulated using Markov Chain Monte Carlo (MCMC) sampling, which indirectly inferred the posterior distribution through computer simulations to find the ordering with the highest probability [20]. A positional variance diagram representing the characteristic ordering of conversions and their uncertainty was derived from the ordering of a set of 1,000,000 MCMC samples. The concept of disease progression modeling can be explained using the analogy proposed by Oxtoby et al., whereby if all patients with a cold have a cough, but only some also experience sneezing, we can strongly estimate that the cough precedes the sneeze [21]. 

The initial analysis was performed to obtain two different subtypes, assuming different fibrosis progression patterns for HBV and HCV. Stage-only modeling, where only a single subtype was modeled, was also performed for each disease. 

Statistical analysis

Continuous variables are shown as medians with interquartile ranges (IQR) and compared using the Wilcoxon rank sum test. Patient sex was expressed as numbers with percentages in parentheses and compared using a chi-square test. Statistical significance was set at p<0.05. All statistical analyses were performed using IBM Corp. Released 2023. IBM SPSS Statistics for Windows, Version 29.0.2.0 Armonk, NY: IBM Corp.

## Results

Patient characteristics

The demographic characteristics and serum and imaging biomarkers are summarized in Table 1. Q-LSC and CEI were significantly lower in the HCV group than those in the control group (p=0.003 and p<0.001, respectively). FIB-4 was significantly higher in the HCV group than those in the HBV and control groups (p=0.01, p<0.001, respectively). Moreover, FIB-4 was significantly higher in the HBV group than that in the control group (p=0.046). APRI was significantly higher in the HBV and HCV groups than those in the control group (p<0.001 and p<0.001, respectively). No other significant differences in biomarkers were observed. The ICC in the raw data of the signal intensities and liver volumes between the two radiologists were as follows: pre-contrast liver, 1.00 (95% CI: 1.00, 1.00); pre-contrast spleen, 0.99 (95% CI: 0.99, 1.00); pre-contrast muscle, 0.99 (95% CI: 0.99, 1.00); post-contrast liver, 1.00 (95% CI: 1.00, 1.00); post-contrast spleen, 0.99 (95% CI: 0.99, 1.00); post-contrast muscle, 0.99 (95% CI: 0.99, 1.00); total liver volume, 1.00 (95% CI: 1.00, 1.00); right liver robe volume, 0.99 (95% CI: 0.99, 1.00).

**Table 1:** Characteristic demographic data and serum and imaging biomarkers Continuous variables are shown as medians with interquartile ranges and compared using the Wilcoxon rank sum test. Patient sex was expressed as numbers with percentages in parentheses and compared using chi-square test. Statistical significance was set at p < 0.05. HBV: hepatitis B virus, HCV: hepatitis C virus, APRI: aspartate aminotransferase to platelet ratio index, FIB-4 index: fibrosis-4 index; CEI: contrast index of liver-muscle signal intensity, Q-LSC: quantitative liver-spleen contrast ratio, RV/TV: right liver lobe volume to total liver volume ratio.

				p-value		
	Control	HBV	HCV	Control vs. HBV	Control vs. HCV	HBV vs. HCV
N	89	29	50			
Age years	65 (31–73)	70 (56–75)	76 (67–80)	0.4	< 0.001	0.01
Male n (%)	41 (46%)	21 (72%)	29 (58%)	0.01	0.2	0.2
Biomarkers						
APRI	0.25 (0.19–0.33)	0.37 (0.23–0.64)	0.42 (0.32–0.71)	< 0.001	< 0.001	0.3
FIB-4 index	1.51 (1.03–2.05)	1.87 (1.13–3.67)	3.16 (1.94–4.16)	0.046	< 0.001	0.01
CEI	1.80 (1.60–1.90)	1.70 (1.45–1.84)	1.63 (1.50–1.76)	0.06	< 0.001	0.4
Q-LSC	2.20 (1.90–2.55)	1.97 (1.84–2.40)	2.05 (1.66–2.22)	0.2	0.003	0.4
RV/TV	0.65 (0.61–0.70)	0.65 (0.55–0.70)	0.60 (0.42–0.69)	0.7	0.06	0.3

Disease progression patterns

**Figure 4:**
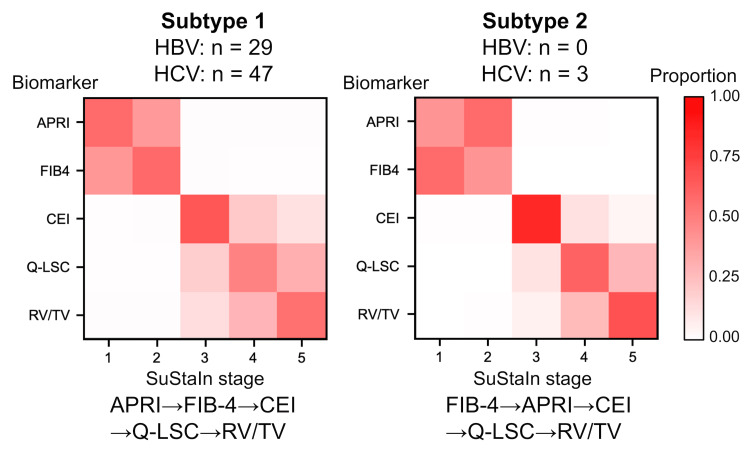
Positional diagrams of the SuStaIn result The image was created by the author. The SuStaIn models of liver fibrosis progression patterns are illustrated using positional variance diagrams. The y-axis of the positional variance diagram shows the biomarkers arranged from top to bottom in order of maximum likelihood sequence in Subtype 1, while the x-axis shows the SuStaIn stage where the conversion of biomarkers appears. The color intensity of each block represents the relative frequency of the Markov Chain Monte Carlo samples ranging from 0 (white) to 1 (red), showing the ordering and uncertainty. Blocks closest to red in the row are considered the most likely position in the ordering. Thus, in the Subtype 1 diagram (left), the blocks closest to red appear on the diagonal. However, strong red blocks with high probability are lacking, indicating relatively weak ordering. Although the initial SuStaIn analysis revealed two liver fibrosis progression patterns, the similarity between them and the fact that most cases belonged to Subtype 1 suggested that Subtype 1 was the best model in either HBV or HCV groups. HBV: hepatitis B virus, HCV: hepatitis C virus, APRI: aspartate aminotransferase to platelet ratio index, FIB-4: fibrosis-4 index, CEI: contrast index of liver-muscle signal intensity, Q-LSC: quantitative liver-spleen contrast ratio, RV/TV: right liver lobe volume to total liver volume ratio.

**Figure 5:**
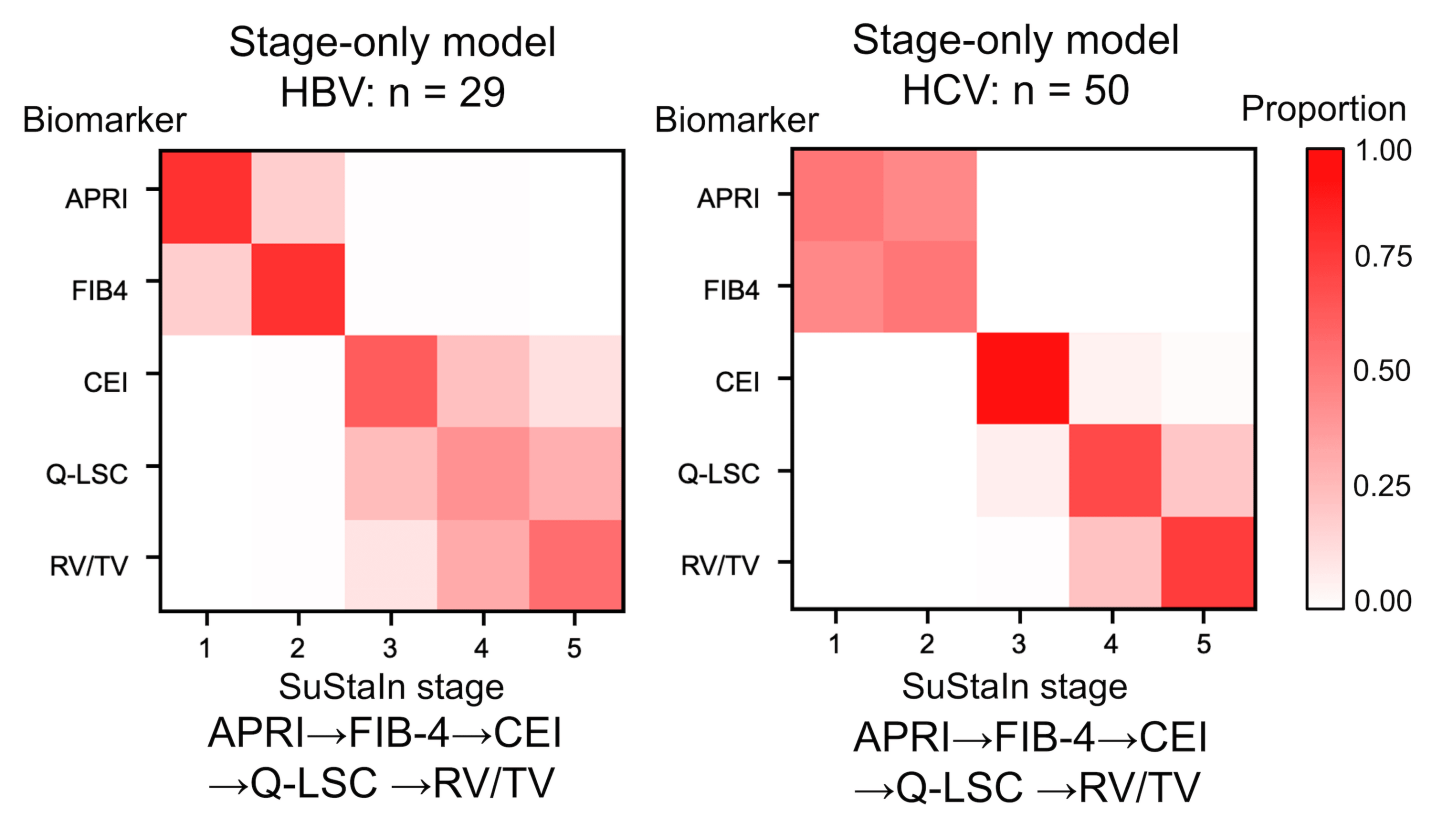
Positional variance diagrams of the stage-only model The image was created by the author. The stages-only models of the liver progression patterns are illustrated using positional variance diagrams. The y-axis shows the biomarkers arranged from top to bottom in order of maximum likelihood sequence, while the x-axis shows the SuStaIn stage where the conversion of biomarkers appears. The color intensity represents the relative frequency of the MCMC samples ranging from 0 (white) to 1 (red), showing the ordering and uncertainty. Blocks closest to red in the row are considered the most likely position in the ordering. In either HBV or HCV groups, liver fibrosis progression patterns are consistent: APRI, FIB-4, CEI, Q-LSC, and RV/TV. Some blocks showed relatively strong red, but there was no row with a single block, indicating that the order of the biomarker conversion could be permuted. However, it was definitely that in liver fibrosis progression, serum biomarker abnormalities (i.e., APRI and FIB-4) preceded imaging biomarker abnormalities (i.e., CEI, Q-LSC, and RV/TV). HBV: hepatitis B virus, HCV: hepatitis C virus, APRI: aspartate aminotransferase to platelet ratio index, FIB-4: fibrosis-4 index, CEI: contrast index of liver-muscle signal intensity, Q-LSC: quantitative liver-spleen contrast ratio, RV/TV: right liver lobe volume to the total liver volume ratio.

**Figure 6:**
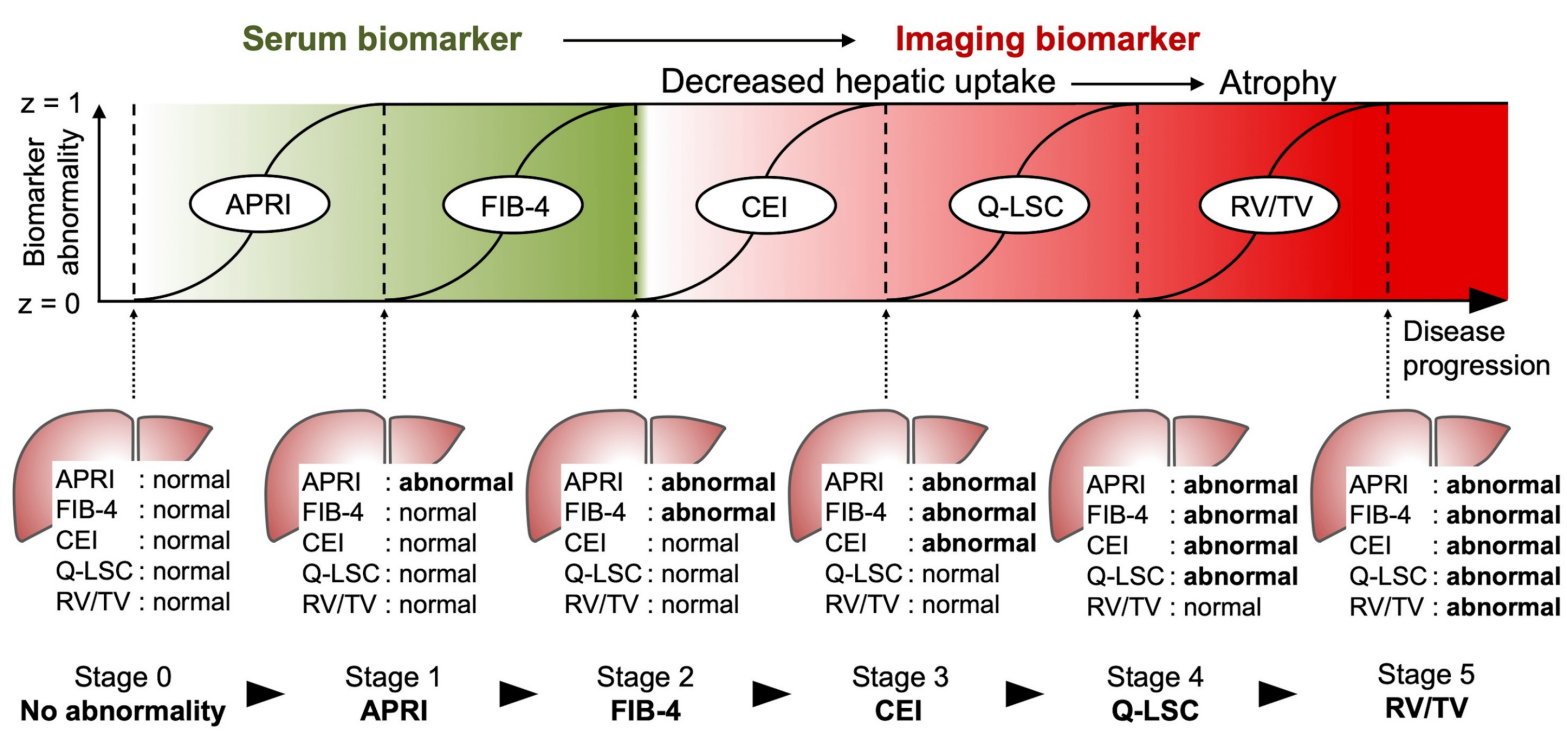
Conceptual overview of the SuStaIn result and biomarker trajectories The image was created by the author. The data-driven biomarker trajectories (top) represent the conversions of biomarker values from a z-score of 0 to 1. This figure helps understand the concept of disease progression and shows each biomarker conversion occurring individually, whereas, in reality, these changes occur simultaneously but at different rates. The bottom row shows snapshots of the status of each biomarker at each stage. APRI: aspartate aminotransferase to platelet ratio index, FIB-4: fibrosis-4 index, CEI: contrast index of liver-muscle signal intensity, Q-LSC: quantitative liver-spleen contrast ratio, RV/TV: right liver lobe volume to the total liver volume ratio.

SuStaIn analysis, which divided the patients into two subtypes, revealed the following disease subtypes: Subtype 1, APRI→FIB-4→CEI→Q-LSC→RV/TV (29 HBV and 47 HCV); and Subtype 2, FIB-4→APRI→CEI→Q-LSC→RV/TV (0 HBV and 3 HCV) (Figure 4). Given that Subtype 1 and 2 were almost identical and most patients were classified under Subtype 1, with only three patients assigned to Subtype 2, Subtype 1 was considered the better disease progression model for all patients in both the HBV and HCV groups. Moreover, in the stages-only model, where all patients were assigned to a single subtype, biomarker conversions in either HBV or HCV groups were identical and ordered as follows: APRI, FIB-4, CEI, Q-LSC, and RV/TV (Figure 5). Therefore, the most likely ordering of the biomarker conversion in liver fibrosis progression in patients infected with either HBV or HCV was determined to be APRI, FIB-4, CEI, Q-LSC, and RV/TV (Figure 6). These subtype progression patterns were plotted using positional variance diagrams, which are two-dimensional histograms showing the positions of each conversion over the MCMC samples. Some blocks showed relatively high likelihood (i.e., strong red), but there was no row with a single red block, so it was possible that the order of the biomarker conversion could be permuted. However, the ordering that in liver fibrosis progression in both the HBV and HCV groups serum biomarker abnormalities (i.e., APRI and FIB-4) were identified at the initial stage, followed by the emergence of imaging biomarker abnormalities (i.e., CEI, Q-LSC, and RV/TV) was definitely.

## Discussion

In this study, the disease progression pattern in liver fibrosis in patients infected with either HBV or HCV was estimated from cross-sectional biomarker data using an unsupervised machine learning tool, SuStaIn. The most likely ordering of the biomarker conversion in liver fibrosis progression was determined to be APRI, FIB-4, CEI, Q-LSC, and RV/TV. To the best of our knowledge, this is the first study to evaluate liver fibrosis progression using data-driven disease progression modeling. While previous studies have evaluated noninvasive alternatives to biopsy for predicting the extent of liver fibrosis, the temporal progression (i.e., the ordering of biomarker conversions) of liver fibrosis has not been well established. A substantial amount of longitudinal data from individual patients is required to clarify the progression pattern of liver fibrosis, but this proves challenging in clinical practice. The strength of SuStaIn lies in reconstructing disease subtypes and stages from only cross-sectional biomarker data snapshots, providing novel insights into liver fibrosis progression. Identifying the disease progression pattern offers several advantages, such as understanding the disease mechanism, accurate patient stratification and prognostication, and stratification for clinical trials. Our results suggest that APRI may be the biomarker that reflects the earliest stage of liver fibrosis. Therefore, among the five biomarkers examined in this study, APRI may be the most suitable for initial screening for liver fibrosis.

It is suggested that serum biomarker conversion occurs in the early stages of liver fibrosis. APRI and FIB-4 serve as effective noninvasive serum biomarkers for predicting liver fibrosis. Notably, the diagnostic performance of APRI in distinguishing between FibroScan results of F0 and F1-F4 was found to be superior to that of FIB-4, whereas the diagnostic performance of APRI in distinguishing between F0-F2 and F3-F4 was equivalent to that of FIB-4 [22]. Another study reported that FIB-4 exhibited better diagnostic performance in distinguishing between F0-F3 and F4 than that of APRI [23]. These findings suggest that the conversion of APRI precedes that of FIB-4 in liver fibrosis progression, which aligns with the results of the present study. However, the ordering of APRI before FIB-4 was not strong, as evidenced by the intermediate color intensities of these blocks in the positional variance diagram of Subtype 1 in SuStaIn. In this regard, a previous report also indicated that FIB-4 and APRI demonstrate nearly equivalent diagnostic performance in distinguishing F0-F1 and F2-F4, as well as F0-F3 and F4 [24]. Increasing the number of patients may resolve this discrepancy. 

In terms of imaging biomarkers, RV/TV has been reported to decrease, particularly in cases of advanced liver fibrosis and cirrhosis. Cirrhosis, which is characterized by right lobe and medial segment atrophy and caudate lobe and lateral segment hypertrophy [25-27], represents the end stage of liver fibrosis and reasonably corresponds to a SuStaIn stage of 5 for RV/TV. CEI and Q-LSC reflect hepatocyte uptake of gadoxetic acid, with decreased CEI and Q-LSC indicating hepatocyte dysfunction [10-12,28]. Hepatocyte dysfunction is expected to occur after hepatocyte damage, which manifests as elevated liver enzymes. Furthermore, CEI demonstrated better diagnostic accuracy than APRI in distinguishing advanced fibrosis stages, such as differentiating F0-3 from F4 [29]. These findings are consistent with the present study's conclusion that serum biomarker conversion precedes imaging biomarker conversion.

This study had several limitations. First, this was a retrospective single-center study with a small sample size. As shown in the positional variance diagrams, the confidence of ordering was not always robust. Furthermore, the clinical validity of the constructed staging model has not been evaluated. Future studies with an increased number of patients are warranted to perform clinical validation. Second, we used a maximum z-score of 1, so the ordering of biomarker conversion from a z-score of 1 to a z-score of 2 or more was not evaluated. Third, magnetic resonance elastography and ultrasound elastography were not included as imaging biomarkers because these imaging modalities are not routinely performed. Fourth, only patients with viral liver diseases were included in this study, limiting the applicability of the inferred liver fibrosis progression patterns to other chronic liver diseases, such as steatotic liver disease. Fifth, there were limitations related to the measurement methods. This study used MRI scanners from multiple vendors, with varying magnetic field correction methods, and ROIs were measured once. We believe that the use of relative values rather than absolute values, the use of z-scores, and the high ICC values have mitigated some of this variability. Sixth, past treatment history for hepatitis virus, viral loads, and duration of viral infection were not considered. Lastly, while this model lacks direct comparison to actual fibrosis severity, it's important to acknowledge that SuStaIn is an unsupervised machine learning method, fundamentally different from supervised machine learning approaches.

## Conclusions

In conclusion, we identified consistent liver fibrosis progression patterns in patients infected with either HBV or HCV using the novel machine learning tool SuStaIn. The data-driven ordering of biomarker conversion was APRI, FIB-4, CEI, Q-LSC, and RV/TV, suggesting that abnormalities in serum biomarkers occurred first, followed by a reduction in hepatic uptake of gadoxetic acid, ultimately leading to atrophy. These findings support prioritizing serum biomarker screening in early fibrosis detection while incorporating imaging to assess disease progression.
